# Childhood trauma and fear of childbirth: findings from a birth cohort study

**DOI:** 10.1007/s00737-023-01328-x

**Published:** 2023-05-27

**Authors:** Elviira Porthan, Matti Lindberg, Juho Härkönen, Noora M. Scheinin, Linnea Karlsson, Hasse Karlsson, Eeva Ekholm

**Affiliations:** 1 FinnBrain Birth Cohort Study, Turku Brain and Mind Center, Department of Clinical Medicine, University of Turku, Turku, Finland; 2 Department of Obstetrics and Gynecology, University of Turku and Turku University Hospital, Turku, Finland; 3 The Hospital District of South Ostrobothnia, Hanneksenrinne 7, 60220 Seinäjoki, Finland; 4 Department of Social Research, Faculty of Social Sciences, University of Turku, Turku, Finland; 5 Department of Political and Social Sciences, European University Institute, Firenze, Italy; 6 Department of Sociology, Stockholm University, Stockholm, Finland; 7 Department of Psychiatry, University of Turku and Turku University Hospital, Turku, Finland; 8 Center for Population Health Research, University of Turku and Turku University Hospital, Turku, Finland

**Keywords:** Childhood trauma, Childhood abuse, Childhood neglect, Fear of childbirth

## Abstract

The aim of this study is to investigate if experiencing childhood trauma (emotional abuse, emotional neglect, physical abuse, physical neglect, or sexual abuse) or a greater total burden of childhood trauma increase the risk of fear of childbirth (FOC). This study included 2556 women living in Southwest Finland. Women were recruited during routine ultrasound visits at gestational week (gwk) 12. Experiencing childhood trauma was assessed in retrospect with the Trauma and Distress Scale (TADS) questionnaire completed at gwk 14. Information on the diagnosis of FOC (ICD-10 diagnosis O99.80) was obtained from the Finnish Medical Birth Register. Associations between childhood trauma (domains and total TADS score) and FOC were analyzed with logistic regression in unadjusted and adjusted models. Emotional abuse (aOR 1.25, 95% CI 1.10–1.42), emotional neglect (aOR 1.26, 95% CI 1.08–1.46), and a greater total burden of trauma (TADS total score) (aOR 1.06, 95% CI 1.02–1.10) increased the risk for FOC. We found no evidence for physical abuse (aOR 1.15, 95% CI 1.00–1.32), physical neglect (aOR 1.06, 95% CI 0.92–1.22), and sexual abuse (aOR 1.24, 95% CI 0.99–1.56) associating with FOC. Childhood emotional abuse, emotional neglect, and a greater total burden of childhood trauma increase the risk for FOC. However, the childhood traumatic events were inquired in retrospect, which could distort the events.

**Supplementary Information:**

The online version contains supplementary material available at 10.1007/s00737-023-01328-x.

## Introduction

Fear of childbirth (FOC) is estimated to affect 5–11% of women (Nilsson et al. 2018; O’Connell et al. 2019). Women with FOC are more likely to prefer cesarean section (CS) as their mode of delivery (Størksen et al. 2015), and up to 45% give birth by CS (Räisänen et al. 2014). Pregnancy is often a stressful time for women with FOC, and FOC can deteriorate family well-being overall (Saisto et al. 2001; Areskog, Uddenberg, and Kjessler 1984).

Several themes have been identified behind fear regarding childbirth, such as lack of trust in healthcare personnel, fear of pain, loss of control, own incompetence, or death or harm to the infant or to oneself (Sjögren 1997; Slade et al. 2019). Nulliparous women may fear the unknown as a result of the lack of experience, whereas in multiparas, fear is more likely to stem from previous negative childbirth experiences (Slade et al. 2019).

Besides negative experiences regarding childbirth, other traumatic events may predispose women to FOC. Childhood trauma is known to associate with a susceptibility to psychiatric disorders, such as mood and anxiety disorders (Carr et al. 2013). Adults who have experienced childhood trauma are less resilient to stress, possibly because of early-life alterations in neurobiological stress regulation systems (Nemeroff 2004). Further, women with a history of childhood trauma and women suffering from FOC have similar personality traits, such as proneness to anxiety (Spice et al. 2009; Mancini, van Ameringen, and MacMillan 1995). This leads us to expect that experience of childhood trauma increases the risk of FOC, although very few previous studies have empirically analyzed the association (Lukasse et al. 2010, 2011; Heimstad et al. 2006).

Childhood trauma can be divided into five domains: emotional, physical and sexual abuse, and emotional and physical neglect (Burgermeister 2007). To date, research has focused on the effects of abuse and two studies have reported that childhood emotional and physical abuse or experiencing any childhood abuse increases the risk for FOC (Lukasse et al. 2010, 2011), whereas the results regarding sexual abuse have been conflicting (Lukasse et al. 2010, 2011; Heimstad et al. 2006). To our knowledge, a possible influence of childhood neglect has not been studied, even though it is more common than abuse. Both emotional and/or physical neglect may impact later psychopathology, as neglect is often long-lasting and usually can be attributed to one’s own family or an otherwise close person.

FOC can lead to decreased family well-being and increased rates of CS. It is important to better understand the reasons behind FOC to find more efficient strategies to prevent and to treat it. Effective treatment of FOC could reduce stress and concerns during pregnancy and lead to more satisfying birth experiences (Uçar and Golbasi 2019). The aim of the current study was to examine if childhood trauma and its different dimensions are associated with FOC. Clarifying the background of FOC helps to focus and develop targeted interventions for FOC.

## Methods

### Study population and data sources

**Fig. 1:**
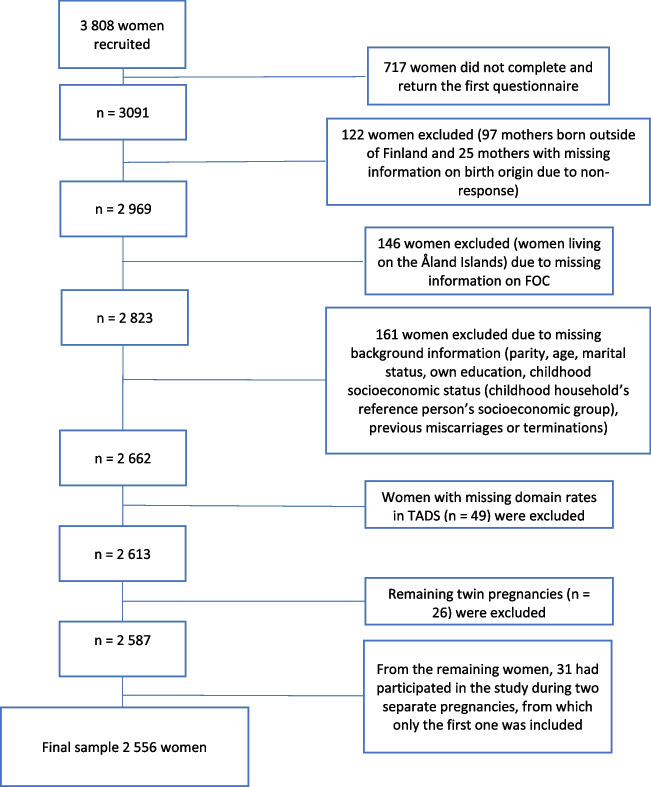
Flow chart of inclusion in the study

This study included 2556 women from the FinnBrain Birth Cohort Study (www.finnbrain.fi). The purpose of the cohort study was to identify biomarkers related to prenatal stress, early life stress exposures, and trajectories for common psychiatric and somatic illnesses. The exclusion process is presented in Fig. 1. A total of 3808 women living in Southwest Finland and the Åland island were recruited between December 2011 and April 2015 during their ultrasound screening visit at gestational week (gwk) 12. The ultrasound screening is offered to all women by public healthcare and is attended with high coverage. The families were approached by the nurses if they had a normal screening result and had sufficient knowledge of Finnish or Swedish. Written consent was received from the participants.

Participants completed the Trauma and Distress Scale (TADS) questionnaire at gwk 14. Questionnaire data was supplemented with data from Statistics Finland and the Finnish Medical Birth Register (FMBR). The study design has been described in detail by Karlsson et al. (2018).

The diagnosis of FOC (ICD-10 diagnosis O99.80) was obtained from the FMBR. All women with the diagnosis were referred for treatment of FOC to the antenatal clinic of a tertiary hospital (Turku University Hospital) mostly by nurses and midwives at maternity clinics in primary care. All women are asked about their fear as a part of routine care on a scale of 0 to 10. This unvalidated screening method is used nationally in maternity clinics in Finland. If the mother is considered as possibly suffering from FOC, she is referred to the tertiary antenatal clinic after gwk 30. They were then assessed in the tertiary antenatal clinic by obstetrician, assistant physician, or midwife and were diagnosed with FOC in the third trimester after a clinical evaluation. After the first visit, the women were offered further appointments to discuss their fear if felt it was needed. Participation in the study did not affect screening or diagnostics.

The domains of childhood trauma (emotional abuse, emotional neglect, physical abuse, physical neglect, and sexual abuse) were assessed with the TADS questionnaire. TADS is a self-reported questionnaire and has been validated in a Finnish sample (Salokangas et al. 2016). Each of the domains consists of 4 to 5 questions (Table S1, Supplementary material). Each question is rated for their frequency in three age categories (0–6, 7–12, and 13–18 years) in a Likert format from 0 to 4 (0 = never, 1 = rarely, 2 = sometimes, 3 = often, and 4 = almost always). The highest rate answered in any of the questions (in any of the age categories) was the rate of the domain. Reversed questions were reverse scored before rating. The TADS total score, representing the total burden of childhood trauma, was calculated as the total score of all five domains.

Information on age, parity, previous miscarriages, and pregnancy terminations was received from the FMBR, and maternal education and childhood socioeconomic status from Statistics Finland. Information on cohabiting status came from the questionnaire. The mothers’ own educational level was assessed in three categories: secondary or lower level education, vocational (lower) tertiary level education (polytechnics and universities of applied sciences), and university education. Childhood socioeconomic status (childhood household’s reference person’s socioeconomic group, i.e., occupation) was divided into four categories: upper level employees, lower level employees, manual workers, and others. Other factors such as intimate partner violence or other traumatic events in adulthood might also have an impact on mental health. We did not have information on these factors. Furthermore, because childhood trauma can affect these adulthood experiences but the opposite influence is not possible, these factors cannot confound the relationship between childhood trauma and FOC.

### Statistical analysis

Correlations between the TADS domains were calculated with Spearman’s rank-order method. The associations between childhood trauma and FOC were explored with logistic regression with separate models, where FOC was the outcome variable and childhood trauma the independent variable. In adjusted models, the chosen covariates were maternal age (as a continuous variable), square of maternal age, parity, and childhood socioeconomic status. The included covariates were factors that precede the diagnosis of FOC or could explain the exposure to childhood traumas. As we did not have information on previous mode of birth or birth experiences, they were not included in the analyses. Age was considered to control for the time after the occurrence of trauma in childhood, as the effect of trauma on later psychopathology could decrease over time (Green et al. 2010). As both younger and older mothers can be at increased risk for FOC (Dencker et al. 2019), the analyses were adjusted also for the square of the age to take into account the possible U-shape of the effect. Childhood socioeconomic status can affect the probability of childhood trauma, as maltreatment is more common in families of low socioeconomic status (Walsh et al. 2019). As earlier birth experiences affect FOC (Størksen et al. 2013), parity was also included in the covariates. Because of the importance of previous birth experiences as a predictor of FOC, analyses were also performed separately to nulli- and multiparous women. Significance levels were set at the 5% level.

The groups of FOC and non-FOC women were also described in terms of other possible covariates (maternal education, cohabiting status, previous miscarriages, and pregnancy terminations). To avoid overcontrolling the analyses, they were used only for characterizing the differences between women with and without FOC and not as covariates in the main analyses.

Statistical analyses were performed with STATA version 14.2.

## Results

The mean age of the subjects was 30.9 years, 52.6% were nulliparous, and 93.5% were cohabiting. Only 2.5% (*n* = 64) of the mothers did not have a post compulsory education degree (included in the category of secondary level education). In total, 165 women (6.5%) had FOC. The proportion of multiparous women in women with and without FOC were 61.2% and 46.5%, consecutively. Characteristics of the women are presented in Table 1. The frequency of each rate in each domain of childhood trauma is presented in Figure S1 (Supplementary material). The correlations between sexual abuse and other domains of trauma were weak, whereas the correlations between emotional abuse, emotional neglect, physical abuse, and physical neglect were stronger (Table S2, Supplementary material).

**Table 1:** Characteristics of the participants

	FOC (*n* = 165)	Non-FOC (*n* = 2391)	*p* value of χ^2^ test
Childhood socioeconomic status			0.011
Upper level	51 (30.9%)	665 (27.8%)	
Lower level	56 (33.9%)	1004 (42.0%)	
Manual worker	34 (20.6%)	532 (22.3%)	
Others	24 (14.6%)	190 (8.0%)	
Education			0.538
Secondary or lower level	67 (40.6%)	884 (37.0%)	
Vocational tertiary level	50 (30.3%)	720 (30.1%)	
Higher level	48 (29.1%)	787 (32.9%)	
Cohabiting status			0.294
Cohabiting	151 (91.5%)	2238 (93.6%)	
Non-cohabiting	14 (8.5%)	153 (6.4%)	
Pregnancy terminations			0.002
0	139 (84.2%)	2149 (89.9%)	
1	17 (10.3%)	201 (8.4%)	
2 or more	9 (5.5%)	41 (1.7%)	
Miscarriages			0.005
0	111 (67.3%)	1869 (78.2%)	
1	43 (26.1%)	409 (17.1%)	
2 or more	11 (6.7%)	113 (4.7%)	
Parity			<0.001
Nulliparous	64 (38.8%)	1280 (53.5%)	
Multiparous	101 (61.2%)	1111 (46.5%)	
Age			0.025
<25	9 (5.5%)	147 (6.2%)	
25–29	29 (17.6%)	672 (28.1%)	
30–34	80 (48.5%)	1000 (41.8%)	
≥35	47 (28.5%)	572 (23.9%)	

The effects of the covariates are presented in Table 2. Only multiparity had a statistically significant association with FOC, whereas maternal age or childhood socioeconomic status had no association with FOC. The associations between childhood trauma and FOC are presented in Table 3. Maternal age or childhood socioeconomic status was not associated with FOC, while multiparity increased the risk (OR 1.72, 95% CI 1.22–2.41, *p* = 0.002). Of the different domains of childhood trauma, emotional abuse (aOR 1.25, 95% CI 1.10–1.42, *p* < 0.001), emotional neglect (aOR 1.26, 95% CI 1.08–1.46, *p* = 0.003), and total TADS sum score (aOR 1.06, 95% CI 1.02–1.10, *p* = 0.003) increased the risk for FOC both in unadjusted and adjusted models. Physical abuse, physical neglect, or sexual abuse was not associated with FOC at the 5% level of significance.

**Table 2:** Odd ratios of covariates for fear of childbirth (*n* = 2556)

Covariates	aOR (95% CI)*	*p* value
Maternal age	1.04 (0.70–1.54)	0.863
Square of maternal age	1.00 (0.99–1.01)	0.900
Parity		
Nulliparous	Ref.	
Multiparous	1.72 (1.22–2.41)	0.002
Childhood socioeconomic status		
Upper level employee	Ref.	
Lower level employee	0.71 (0.48–1.06)	0.091
Manual worker	0.81 (0.51–1.27)	0.349
Others	1.39 (0.72–2.68)	0.326

*All variables were included in the same model

**Table 3:** Odd ratios for fear of childbirth

Childhood trauma*	OR	*p* value	aOR†	*p* value
Emotional abuse	1.25 (1.10–1.41)	<0.001	1.25 (1.10–1.42)	<0.001
Emotional neglect	1.26 (1.09–1.46)	0.002	1.26 (1.08–1.46)	0.003
Physical abuse	1.14 (0.99–1.31)	0.065	1.15 (1.00–1.32)	0.055
Physical neglect	1.06 (0.92–1.21)	0.419	1.06 (0.92–1.22)	0.403
Sexual abuse	1.22 (0.98–1.52)	0.077	1.24 (0.99–1.56)	0.061
Total TADS sum score	1.06 (1.02–1.10)	0.004	1.06 (1.02–1.10)	0.003

*Analyses for domains of childhood trauma and total TADS score were performed in separate models

†aOR adjusted for maternal age, square of maternal age, parity, and childhood socioeconomic status

Emotional abuse was associated with FOC in both nulli- and multiparous women whereas emotional neglect and a greater total burden of trauma (total TADS score) were associated with FOC only in nulliparous women. Physical abuse, physical neglect, or sexual abuse was not associated with FOC (Table S3, supplementary material).

## Discussion

### Main findings

Experiencing emotional abuse, emotional neglect, and a greater total burden of trauma in childhood were associated with an increased risk of FOC. Interestingly, we found no association between sexual abuse, physical abuse, or physical neglect and FOC.

In subpopulation analyses (nulli- and multiparous women separately), emotional abuse increased the risk for FOC both in nulli- and multiparous women, whereas emotional neglect and greater total burden of trauma only in nulliparous women.

### Interpretation

Our results regarding emotional abuse were in line with some previous reports (Lukasse et al. 2010, 2011), whereas the results on physical and sexual abuse are in conflict with some previous ones (Lukasse et al. 2010, 2011; Heimstad et al. 2006). However, there were multiple differences in the study designs such as clinical evaluation for FOC in our study whereas questionnaires were used in previous ones. Therefore, our study is not entirely comparable to previous ones. To date, this is the first study to investigate if emotional or physical neglect affects FOC. The prevalence of FOC in this sample (6.5%) was similar to previous findings of 5–11% (Nilsson et al. 2018; O’Connell et al. 2019).

Physical abuse did not increase the risk for FOC. Previous studies have reported an increase in FOC in association with childhood physical abuse, both in unadjusted and adjusted models (Lukasse et al. 2010, 2011). Differences in the questionnaires used may partially account for the discrepant results. The TADS may depict a somewhat more serious degree of physical abuse than the Norvold Abuse Questionnaire used in previous studies (Lukasse et al. 2010, 2011). In addition, the association between FOC and physical abuse fell only slightly short of the 5% level of significance.

Childhood sexual abuse was not associated with FOC. Previously, childhood sexual abuse was associated with FOC in nulliparous but not in multiparous women (Lukasse et al. 2010). Another study conducted in multiparous women showed an increased risk for FOC only in an unadjusted model, but not when adjusted for age, education, marital status, adult abuse, and mental distress (Lukasse et al. 2011). As sexual abuse was reported much less frequently than other domains of childhood trauma, our sample size might have been too small to observe the association.

Experiencing childhood trauma has been associated with decreased resilience later in life (Beutel et al. 2017). Trauma during the programming of the central nervous system may change stress regulation neurobiology, and these early-life alterations could result in decreased resilience in adulthood (Nemeroff 2004). In turn, decreased resilience might play a part in FOC. Personality traits often associated with a history of childhood trauma might also contribute to FOC. Indeed, women with FOC as well as women who have experienced childhood trauma often have similar personality traits, such as an anxiety-prone personality (Spice et al. 2009; Mancini, van Ameringen, and MacMillan 1995). Women with low resilience and a more anxiety-prone personality might find it more difficult to cope with unpredictability and pain associated with childbirth and, thus, feel incapable to give birth, leading to increased levels of FOC.

Emotional abuse increased the risk for FOC both in nulli- and multiparous women, whereas emotional neglect and a greater total burden of childhood trauma increased the risk only in nulliparous women. In a previous study, the effects of emotional abuse and experiencing any abuse on FOC were smaller in multiparous women, although the risk was increased in both parity groups (Lukasse et al. 2010). This different effect of childhood trauma on nulli- and multiparous women might reflect that women with proneness to anxiety might feel fear before their first labor due to lack of experience, but feel more confidence during the subsequent pregnancies as they already have experience of the childbirth. Moreover, in multiparous women, the previous birth experiences can affect the expectations and fear regarding childbirth (Saisto, Ylikorkala, and Halmesmäki 1999). Accordingly, a previous study found an association between anxiety-prone personality and FOC only in nulliparous women (Jokić-Begić, Žigić, and Radoš 2014).

Our results show that childhood trauma is related to FOC. Previous research on the subject is scarce and our study considers, besides abuse, also neglect. On the basis of the present results, history of childhood trauma should be recognized in maternity health care. The effect of childhood trauma was stronger in nulliparous women, and therefore they could especially benefit from screening of childhood traumas when treated for FOC. Adults who have been traumatized in their childhood are prone to have a more insecure attachment style (Fuchshuber et al. 2019), may find it difficult to depend on others, and consequently, have more difficulties in creating and preserving trust in health care personnel. Women with FOC often have challenges trusting, besides their own coping during birth, also health care personnel managing their prenatal care and delivery (Slade et al. 2019; Sjögren 1997). Of the covariates, multiparity appeared to be an important factor suggesting previous birth experiences should be paid attention to.

### Strengths and limitations

One of the strengths of our study was that the women who were diagnosed with FOC suffered from clinically severe FOC. We were able to link information from two national registers to our data, and therefore, to use information from multiple sources. However, some limitations should be acknowledged. As childhood traumatic events were inquired in retrospect, they could be recalled falsely. Current FOC could distort the recalled experiences to be more negative. This is a well-acknowledged limitation in studies using retrospective self-reports. On the other hand, this approach is the only feasible means of assessing childhood adversity retrospectively, as, e.g., interviews would be not only laborious to carry out, but also expected to suffer from the same biases.

Previous negative birth experiences and adverse pregnancy outcomes can increase FOC. Therefore, it would have been interesting to know how these might have affected the results. Unfortunately, no information on previous birth experiences, outcomes, or previous FOC diagnoses were available. In addition, we did not have information on other potentially traumatic adulthood events, such as intimate partner violence, or all mental health disorders such as post-traumatic stress disorder that may affect FOC. However, because these factors can be affected by childhood trauma, rather than the opposite, they cannot be considered as confounders.

As nulli- and multiparous women have different reasons behind their FOC, we performed the analyses, besides to the whole population, also separately to nulli- and multiparous women. However, as the number of primiparous women with FOC was small, the results should be interpreted with caution.

## Conclusions

Emotional abuse, emotional neglect, and a greater total burden of childhood trauma increased the risk for FOC after taking into account maternal age, childhood socioeconomic status, and parity. Recognizing the special characteristics of women with a history of trauma could help to develop more individualized and effective interventions. Further, information on women’s history of childhood trauma could help encounter and understand them better. Prevention of childhood neglect and abuse could decrease FOC as well as other psychical burden throughout the life. Information on childhood traumatic events was inquired in retrospect and therefore the events could be recalled falsely. This should be taken into account when interpreting our results.

## Supplementary information

ESM 1 (DOCX 33 kb)
