# Supplementary material for: Childhood trauma and fear of childbirth: findings from a birth cohort study
Source: Arch Womens Ment Health. 2023 May 27;26(4):523–9. doi: 10.1007/s00737-023-01328-x (PMC10333423; doi:10.1007/s00737-023-01328-x)
Supplement: Supplementary file 1 — (DOCX 33 kb) [file 737_2023_1328_MOESM1_ESM.docx]

**Table S1. Questions contributing to each of the TADS domains**

| Factor |  |
| --- | --- |
| Emotional abuse | 10. I felt rejected by my parents/caregivers  12. When I was young, I was humiliated by people in my family  14. I believe that I am a bad person  26. When I was young, I felt hated by a member or members of my family  32. I feel that I was put down, criticised, and made to feel inferior when I was young |
| Emotional neglect | 5. When I was young, I felt valued or important (r)  8. My family was emotionally warm and loving (r)  13. When I was young, my family looked after each other (r)  21. I respect myself (r)  40. My family was supportive and encouraging when I was young (r) |
| Physical abuse* | 9. When I was young, I was hit so hard that it left marks, cuts, or bruises  16. I have experienced serious physical assault  20. I think I was physically abused when I was young  24. I have been involved in life-threatening situations  42. I felt afraid of someone in my family |
| Physical neglect* | 1. When I was young, I felt safe and protected by someone (r)  4. I often wear ragged or dirty clothes to school  6. My parents/caregivers were often drunk, stoned, or wasted  31. If I needed treatment someone would always take me to see a doctor or nurse when I was young (r) |
| Sexual abuse | 22. When I was young, someone touched me or tried to make me touch them in a sexual way  25. I was forced to keep secrets about someone sexually interfering with me when I was young  30. I have experienced sexual assault  33. Someone sexually molested me when I was young  41. I believe that I was sexually used when I was young |
| (r) = reversed before rating  * Contrary to the original TADS, physical abuse domain question 17 (Adults noticed cuts, bruises, or marks from when I was beaten) was replaced with question 42 and the physical neglect domain question 2 (When I was young, I was often hungry) was removed. This increased the internal consistencies of the domains in a Finnish sample(Salokangas et al. 2016) | |

**Table S2. Spearman correlations between domains of TADS**

|  | Emotional neglect | Physical abuse | Physical neglect | Sexual abuse |
| --- | --- | --- | --- | --- |
| Emotional abuse | 0.62 | 0.57 | 0.50 | 0.24 |
| Emotional neglect |  | 0.48 | 0.55 | 0.21 |
| Physical abuse |  |  | 0.48 | 0.27 |
| Physical neglect |  |  |  | 0.22 |

All p-values < 0.001

**Table S3. Odd Ratios for Fear of Childbirth in nulliparous and multiparous women**

| **Childhood trauma*** | Nulliparous | p-value | Multiparous | p-value |
| --- | --- | --- | --- | --- |
| **Emotional abuse** |  |  |  |  |
| OR | **1.33 (1.09-1.62)** | **0.005** | **1.20 (1.02-1.40)** | **0.028** |
| aOR† | **1.32 (1.09-1.62)** | **0.006** | **1.20 (1.02-1.41)** | **0.030** |
| **Emotional neglect** |  |  |  |  |
| OR | **1.37 (1.09-1.73)** | **0.008** | 1.16 (0.95-1.42) | 0.140 |
| aOR† | **1.38 (1.09-1.74)** | **0.008** | 1.17 (0.96-1.44) | 0.120 |
| **Physical abuse** |  |  |  |  |
| OR | 1.24 (1.00-1.53) | 0.053 | 1.08 (0.90-1.30) | 0.412 |
| aOR† | 1.23 (0.99-1.53) | 0.056 | 1.09 (0.90-1.31) | 0.380 |
| **Physical neglect** |  |  |  |  |
| OR | 1.10 (0.89-1.36) | 0.382 | 1.02 (0.86-1.22) | 0.814 |
| aOR† | 1.11 (0.89-1.37) | 0.348 | 1.03 (0.86-1.23) | 0.779 |
| **Sexual abuse** |  |  |  |  |
| OR | 1.31 (0.95-1.80) | 0.096 | 1.16 (0.85-1.58) | 0.342 |
| aOR† | 1.33 (0.96-1.84) | 0.091 | 1.15 (0.84-1.57) | 0.380 |
| **Total TADS score** |  |  |  |  |
| OR | **1.08 (1.02-1.14)** | **0.009** | 1.04 (0.99-1.09) | 0.131 |
| aOR† | **1.08 (1.02-1.15)** | **0.009** | 1.04 (0.99-1.10) | 0.121 |

* Analyses for domains of childhood trauma and total TADS score were performed in separate models

† adjusted for childhood socioeconomic status, maternal age and square of maternal age

**Figure S1. Frequency (% of the study population) of different domains of childhood maltreatment**
